# Contributions of Gene Modules Regulated by Essential Noncoding RNA in Colon Adenocarcinoma Progression

**DOI:** 10.1155/2020/8595473

**Published:** 2020-03-20

**Authors:** Chunhua Li, Xiaorong Yu, Jianping Lu, Liyu Zheng, Dahua Xu, Zelong Xu, Liqiang Wang, Ying Cui, Yeshuang Li, Hong Wang, Jiankai Xu, Kongning Li

**Affiliations:** ^1^College of Bioinformatics Science and Technology, Harbin Medical University, Harbin, Heilongjiang Province 150081, China; ^2^Key Laboratory of Tropical Translational Medicine of Ministry of Education, Hainan Medical University, Haikou, Hainan Province 571199, China

## Abstract

Noncoding RNAs (ncRNAs), especially microRNA (miRNA) and long noncoding RNA (lncRNA), have an impact on a variety of important biological processes during colon adenocarcinoma (COAD) progression. This includes chromatin organization, transcriptional and posttranscriptional regulation, and cell-cell signaling. The aim of this study is to identify the ncRNA-regulated modules that accompany the progression of COAD and to analyze their mechanisms, in order to screen the potential prognostic biomarkers for COAD. An integrative molecular analysis was carried out to identify the crosstalks of gene modules between different COAD stages, as well as the essential ncRNAs in the posttranscriptional regulation of these modules. 31 ncRNA regulatory modules were found to be significantly associated with overall survival in COAD patients. 17 out of the 31 modules (in which ncRNAs played essential roles) had improved the predictive ability for COAD patient survival compared to only the mRNAs of those modules, which were enriched in the core cancer hallmark pathways with closer interactions. These suggest that the ncRNAs' regulatory modules not only exhibit close relation to COAD progression but also reflect the dynamic significant crosstalk of genes in the modules to the different malignant extent of COAD.

## 1. Introduction

Colon adenocarcinoma (COAD) is a common tumor of the digestive system, and its incidence and fatality rate are increasing in recent years [1]. The progression of COAD is the major cause of serious morbidity and mortality in cancer patients [2]. In most cases, low-stage COAD (stages I and II) are curable by surgical resection, and about 70% of stage III COAD cases with regional lymph node metastasis are curable through a combination of surgery and adjuvant chemotherapy. Despite the improved survival rate from recent advances in chemotherapy and target agents, advanced metastatic COAD (stage IV) remains largely incurable [3, 4]. Thus, there is a great urgency to understand the key molecular biomarkers involved in COAD metastasis and identify these biomarkers for COAD malignancies, as well as the prognostic markers for patient survival.

Protein-coding genes account for only a small portion of the human genome, whereas more than 98% of transcripts consist of noncoding RNAs [5–7]. The increased sensitivity of experimental assays had revealed that noncoding RNAs (ncRNAs) have impacts on a variety of important biological processes, particularly microRNA (miRNA) and long noncoding RNA (lncRNA) [8–10]. Numerous studies have demonstrated that miRNA and lncRNA play a vital biological role in regulating COAD processes [11, 12]. MiRNAs are 18 nucleotides to 25 nucleotides in length. They play a central role as master regulators of gene expression at the posttranscriptional level. Previous studies have discovered that the growth and migration of COAD are greatly influenced by miRNAs [13–15]. Long noncoding RNAs (lncRNAs) are a class of pervasively transcribed RNA molecules. They have a length of more than 200 nucleotides and do not encode proteins [16]. Many evidences indicate that lncRNAs could play a critical role in regulation in cellular processes, such as cancer progression and metastasis, through their influences on miRNAs. For example, lncRNA BACE1AS can inhibit miR-485-5p resulting in alleviating the inhibition of BACE1 in COAD [17] and lncRNA HAGLROS plays a sponge role to inhibit miR-100 for the regulation of apoptosis and autophagy in COAD cells [18]. Collectively, ncRNAs play a significant role in the life cycle of COAD progression. Therefore, it is urgent to decipher the mechanism of COAD progression more comprehensively, which incorporates not only the genes but also ncRNAs at the posttranscriptional level.

COAD transformation from the normal colonic mucosa develops through a progressive accumulation of molecular and physiological changes. The continuous changes of gene expression drive the COAD from a low stage (stage I and stage II) to a high stage (stage III and stage IV), and ncRNA has an irreplaceable effect on the regulation of gene expression. Consequently, it is necessary to mine the ncRNA regulatory gene modules that accompany the COAD progression and explore the underlying molecular links across different pathological stages in order to screen the potential prognostic biomarkers for COAD. In this study, we introduced a multidimensional integration strategy based on gene expression profiling, miRNA and lncRNA expression profiling, protein-protein interactions (PPIs), and posttranscriptional regulation data to identify gene modules that are biologically relevant, along with their ncRNA regulators involved in COAD progression [19]. Systematic construction and analysis of these gene modules and their ncRNA regulators across different pathological stages can elucidate the mechanism of COAD progression from a comprehensive view of genomics and ncRNA regulation. It can also identify the biomarkers of COAD malignancies and prognostic markers of patient survival.

## 2. Materials and Methods

### 2.1. Data Resources

The expression dataset of 249 patients for COAD were obtained from the TCGA, among which 7 patients lacked staging information. The level 3 gene expression datasets were used to map and summarize gene level (RPKM) RNA-seq [20]. Genes with RPKM expression values of 0 were removed from all samples. For logarithmic transformation, RPKM expression with no gene values was set to 0.05. The RNA-seq gene expression values were transformed in terms of log2. As a result, the expression levels of 20,036 genes were obtained. The same process was performed for the miRNA expression dataset, and the expression levels of 2104 miRNAs were obtained. In accordance to the reannotation of the mRNA expression dataset, the expression of 1657 lncRNAs was acquired [21].

Three independent datasets (GSE29623, GSE39582, and GSE17536) were used to confirm the contribution of the modules to COAD patients' survival, covering 65, 579, and 177 mRNA microarrays.

CLIP-seq is the crosslinking of immunoprecipitation and high-throughput sequencing. It is a method used in molecular biology to combine UV crosslinking with immunoprecipitation in order to analyze protein interactions with RNA or to precisely locate RNA modifications [22, 23]. CLIP-based techniques can be used to map RNA binding sites in proteins or RNA modification sites [24, 25]. Regulatory interactions of miRNA-target relationships were downloaded from starBase v2.0 [26]. 606,048 miRNA-mRNA interactions and 10,231 miRNA-lncRNA interactions were collected.

### 2.2. Identifying COAD-Related Differentially Expressed Genes (DEGs)

Differential expression analysis was used to filter genes involved in the malignant progression of COAD. This was achieved by, respectively, comparing the gene expression levels in stage III or stage IV COAD with those in low-stage COAD (stage I and stage II COAD). Differential expression was detected by R package SAM [27]. It was determined that stage III and stage IV COAD-DEGs had a 5% false discovery rate.

### 2.3. Generating Stage III and Stage IV COAD-Related Functional Gene Modules

The database STRING (Search Tool for the Retrieval of Interacting Genes/Proteins) is dedicated to protein-protein interactions (PPI). Among currently available databases, it provides the most comprehensive view on PPIs and thus acts as a metadatabase for extensive PPI analysis [28]. A PPI network containing 9061 proteins and 69,400 high-confidence interactions with a score cutoff of 0.9 was extracted. Then, stage III and stage IV COAD-DEGs were mapped onto the PPI network. The maximal connected components (MCCs) which contain the DEGs and the neighboring nodes of DEGs were subsequently obtained.

Based on the MCCs generated above, stage III and stage IV COAD-related functional gene modules were mined, via applying a well-developed MCODE method with default parameters [29].

### 2.4. Determining Significant Crosstalk Module Pairs of Stage III and Stage IV COAD

Based on the assumption that the crosstalk between the stage III and stage IV COAD-related modules is significant (when the number of their interactions is significantly more than random distribution), 1000 random PPI networks were constructed (with the degree distribution of nodes in the original network remaining unchanged) [30, 31]. For each pair of stage III and stage IV COAD-related modules, the real number of interactions between the module pair and the random distribution extracted from 1000 random PPI networks was compared. The *P* value was computed as follows:

(1) $$\begin{array}{c} P=\frac{\sum_{i=1}^{N}S_{i}}{N}. \end{array}$$

When *S*_*i*_ = 1, it represents that the number of random interactions between the two modules was more than the real one; otherwise *S*_*i*_ = 0 [32]. The crosstalk between each module pair was defined as significant if it had a *P* value less than 0.05.

### 2.5. Construction of the COAD-Related ncRNA Posttranscriptional Regulatory Network by Integrative Computational Method

To identify gene modules and their essential ncRNAs that are likely to play important roles during COAD malignant progression, an integrated approach was adopted to construct the COAD-related ncRNA posttranscriptional regulated network. To identify the regulations from miRNAs to targets, both the regulatory interactions and the inverse expression relationships between miRNAs and targets in the context of COAD progression were combined. The active miRNA-target pairs were identified by the Ago CLIP-supported regulatory data in COAD, and their Pearson correlation coefficients (*R*) were computed. All of the candidate miRNA-target pairs with *R* < 0 and FDR < 0.05 were identified as active miRNA-target interactions. To evaluate the regulations from lncRNAs to genes, a two-stage analysis method was explored. First, a hypergeometric test was conducted to compute the significance of shared miRNAs for each lncRNA-gene pairs. And lncRNA-gene pairs with *P* values < 0.05 were considered as candidate lncRNA-gene interaction pairs. Then, in order to identify the active lncRNA-gene pairs in COAD, the Pearson correlation coefficient (*R*) was computed for each candidate lncRNA-gene pair identified above. All the candidate lncRNA-gene pairs with *R* > 0 and *P* adjusted < 0.05 were identified as active lncRNA-gene interactions. After assembling all identified lncRNA-gene interactions, miRNA-target interactions, and PPI interactions, the COAD-related ncRNA posttranscriptional regulatory network was generated.

Pivot analysis was conducted to identify the microRNAs and lncRNAs which significantly regulated both of the significant crosstalks of the stage III and stage IV COAD-related module pair [33]. It was required that the number of regulations between each regulator and each module pair was more than two; meanwhile, a significant proportion of its targets enriched in each module determined by the hypergeometric test had a *P* value less than 0.05.

### 2.6. Survival Analysis of ncRNA Regulatory Modules

Multivariable Cox regression analysis was used to evaluate the association between survival and the expression level of each ncRNA regulatory module. A positive regression coefficient indicated that an increased expression is associated with an increased risk of survival (risk factors); conversely, a negative value indicated that increased expression is associated with a decreased risk of survival (protective factors). More specifically, a risk score was assigned to each patient, in accordance to a linear combination of the expression levels of the ncRNA regulatory module factors, weighted by the regression coefficients from the aforementioned unilabiate Cox regression analysis. The risk score for each patient was calculated as follows:

(2) $$\begin{array}{c} \text{Risk}\_\text{score}=\overset{n}{\underset{i=1}{\sum }}\beta_{i}*{\text{Exp}}_{\text{factor}\left(i\right)}, \end{array}$$

where *β*_*i*_ is the Cox regression coefficient for the *i*th factor of the ncRNA regulatory module and *n* is the number of factors in the ncRNA regulatory module. All patients were thus dichotomized into high-risk and low-risk groups, with the median risk score as the cutoff point. Patients with higher risk scores were expected to have poor survival outcomes. The Kaplan-Meier method was further used to estimate the overall survival time for the two groups. The differences in the survival times were analyzed using the log rank test.

### 2.7. Functional Enrichment Analysis and Construction of Hallmark Pathway Network

To explore the functional roles of the modules, the genes of modules were used to perform hallmark pathway functional enrichment. This process was achieved by the use of hypergeometric analysis. If two hallmark pathways were significantly enriched in the same COAD-related ncRNA regulatory modules, then there may be links between these two hallmark pathways in the process of COAD progression. Based on this assumption, through a hypergeometric test, these potentially linked hallmark pathways were recognized and COAD-related hallmark pathway networks were constructed.

## 3. Results

### 3.1. Determination of Low-Stage COAD Group and Differential Expression Analysis of Different Stages of COAD

Based on level 3 gene expression profiles and clinical information of COAD from the TCGA database, the team applied the overall survival analysis for COAD patients in different pathological stages. No significant differences were observed for overall survival analysis between stage I and stage II COAD patients. SAM [27] was applied to identify differentially expressed genes (DEGs) between stage I and stage II COAD patients and to identify any significant differences in the gene expression between these patients. Again, no significant differences were found between stage I and stage II COAD patients. Therefore, stage I COAD and stage II COAD samples were merged as the low-stage COAD group for the following analysis.

**Figure 1:**
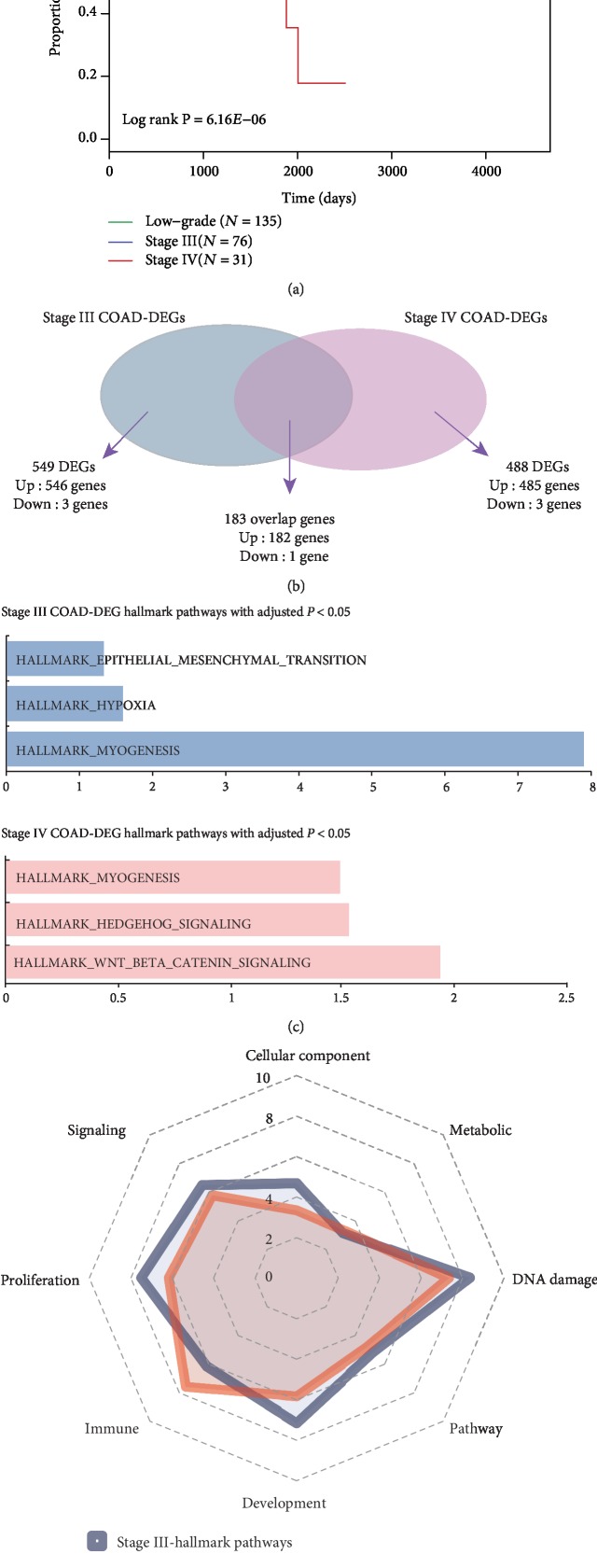
The survival analysis of different stages of COAD and function enrichment analysis of COAD-related DEGs and modules. (a) The Kaplan-Meier method was used to estimate the overall survival time for the three COAD groups (low-stage COAD group, stage III COAD group, and stage IV COAD group). The differences in the survival times were analyzed using the log rank test. The *P* value for survival analysis was 6.16*E*‐06. (b) Distributions of stage-III COAD-DEGs, stage-IV COAD-DEGs, and their overlap DEGs. DEGs were identified by SAM analysis by comparing the stage III COAD or stage IV COAD to the low-stage COAD samples at a FDR cutoff of 0.05. (c) Enrichment results for hallmark pathway analysis of stage III COAD-DEGs or stage IV COAD-DEGs. The information of the hallmark pathway was obtained from the MSigDB database. Pink corresponds to stage III DEGs, and blue corresponds to stage IV DEGs. (d) Trends of hallmark pathway process category enrichment for stage III and stage IV COAD-related modules. The orders of categories of the hallmark pathway listed in the radar diagram are based on the normalized number of their enriched modules. Pink corresponds to trends of hallmark pathway category enrichment results of stage III COAD-related modules, and blue corresponds to the stage IV COAD-related modules.

COAD samples were then divided into three groups: the low-stage COAD group, the stage III COAD group, and the stage IV COAD group. Survival analysis showed that there are significant differences among the three groups (*P* = 6.16*E*‐06). In order to observe whether there are significant differences in gene expression levels among the three COAD groups, the stage III COAD-DEGs were computed by comparing the stage III COAD and low-stage COAD samples at a FDR cutoff of 0.05. The same procedure was also applied to the identification of stage IV COAD-DEGs. In total, 732 stage III COAD-DEGs and 671 stage IV COAD-DEGs were obtained (Figure 1).

The Molecular Signatures Database (MSigDB) is one of the most widely used knowledge-based repositories of annotated sets of genes involved in biochemical pathways, signaling cascades, expression profiles from research publications, and other biological concepts [34]. Through a combination of automated approaches and expert curation, MSigDB developed a collection of “hallmark” gene sets to provide more refined and concise inputs for gene set enrichment analysis. The team then focused on the stage III COAD-DEGs and stage IV COAD-DEGs in the context of cancer hallmark gene sets (Figure 1). Based on hallmark functional analysis of stage III COAD-DEGs and stage IV COAD-DEGs, it was observed that the stage III COAD-DEGs tend to be significantly enriched in “MYOGENESIS,” “HYPOXIA,” and “EPITHELIAL_MESENCHYMAL_TRANSITION,” while stage IV COAD-DEGs tend to be significantly enriched in “WNT_BETA_CATENIN_SIGNALING,” “HEDGEHOG_SIGNALING,” and “MYOGENESIS.” The result suggests that trends of hallmark functions for different stages of COAD-DEGs were different.

### 3.2. Identification of Functional Stage III and Stage IV COAD-Related Modules

In addition to hallmark functional analysis of stage III and stage IV COAD-DEGs, the stage III and stage IV COAD-related PPI subnetworks were obtained via mapping said COAD-DEGs to the PPI network, respectively [28]. In order to identify functional stage III and stage IV COAD-related modules, MCODE [29] was applied to compute the modules from stage III and stage IV COAD-related PPI subnetworks. As a result, 83 stage III- (Supplementary [Supplementary-material supplementary-material-1]) and 79 stage IV-related (Supplementary [Supplementary-material supplementary-material-1]) functional modules were obtained.

Since the trends of hallmark functional analysis for DEGs in different COAD stages were different, the same analysis for COAD-related modules in different stages was also performed to identify any significant differences. Based on functional analysis, no significant differences were discovered in the trends of hallmark functional analysis for COAD-related modules in different stages. Results showed that the hallmark process category “metabolic” was enriched by fewer COAD-related modules in both stage III and stage IV. This indicated that the activity of the process category “metabolic” does not have significant changes in relation to COAD progression. However, high activities were observed in stage III and stage IV COAD for three hallmark process categories, which were “DNA damage,” “signaling,” and “proliferation.” It was also discovered that hallmark process category “development” and “proliferation” had high activities in stage III. This indicated that the activities of process category “development” and “proliferation” were upregulated when low-stage (stage I and stage II) COAD transformed into stage III COAD, and activities of process category “development” and “proliferation” were downregulated when transformation from stage III COAD into stage IV COAD was in progress. It was also found that the process category “immune” was more active in stage IV COAD than stage III COAD. This indicated that the process category “immune” was upregulated when transformation from stage III COAD into stage IV COAD was in progress.

### 3.3. Coad-Related Gene Modules and Their ncRNA Regulators' Contribution to COAD Survival

The increased sensitivity of experimental assays has revealed that ncRNAs, especially miRNA and lncRNA, impact a variety of important biological processes via posttranscriptional regulation [13, 15]. A lot of evidence suggests that miRNA and lncRNA play important roles in COAD progression [11, 12]. To identify gene modules and their essential ncRNAs that are likely to play an important role in COAD malignant progression, an integrated approach was adopted to construct COAD-related ncRNA posttranscriptional regulatory networks. This includes 353 miRNAs, 126 lncRNAs, 9061 mRNAs, and 135,309 edges.

COAD transformation from the normal colonic mucosa arises through progressively accumulated changes. Along with the progression of COAD, COAD-related genes of different pathological stages of COAD are also continuously changing. Significant crosstalk module pairs of stage III and stage IV COAD could reflect the progressive accumulation of COAD-related gene changes. Significant crosstalk module pairs are likely to share some common ncRNA regulators at the posttranscriptional level. To explore these significant crosstalk module pairs and how their ncRNA regulators contribute to COAD survival, the significant crosstalk module pairs were first determined based on a permutation test. Then, a pivot analysis was applied on the currently curated ncRNA posttranscriptional regulations in order to identify the ncRNA regulators of these module pairs. 40 significant crosstalk module pairs (i.e., 40 new modules) that shared common ncRNA regulators at the posttranscriptional level were obtained as a result.

**Figure 2:**
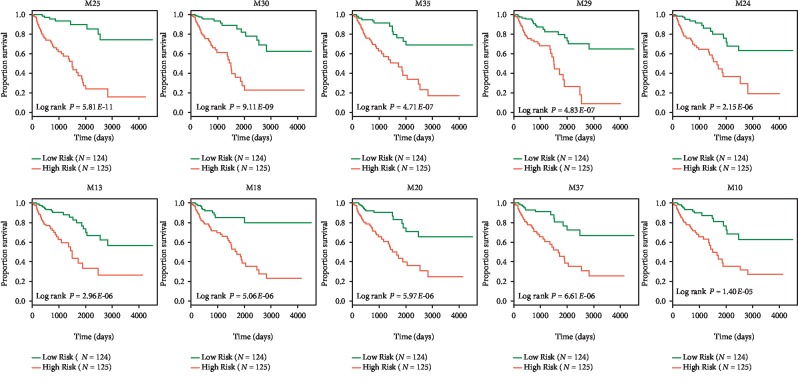
Top ten ncRNA-regulated modules effectively predict the survival of COAD patients. The Kaplan-Meier method was used to estimate the overall survival time for the all the 40 ncRNA-regulated modules. The differences in the survival times of each ncRNA-regulated module were analyzed using the log rank test.

In order to determine whether these ncRNA-regulated modules contribute to COAD survival, for each module, its predictive ability was evaluated for the survival of COAD patients (as described in the Materials and Methods). Notably 31 out of the 40 modules were found to be significantly associated with the overall survival of COAD patients (Figure 2, Supplementary [Supplementary-material supplementary-material-1]). It can be clearly seen that the modules regulated by ncRNAs not only exhibit a close relation to COAD progression but also reflect the dynamic significant crosstalk of module genes to a different malignant extent of COAD.

### 3.4. COAD-Related ncRNAs Regulate Core Hallmark Pathways

**Figure 3:**
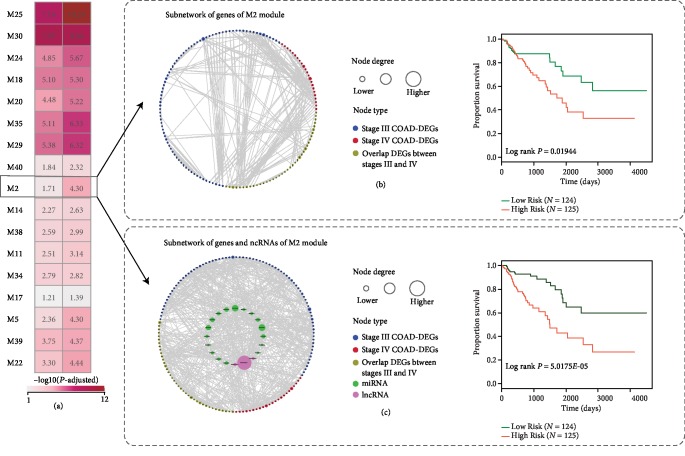
The comprehensive view of survival analysis features of group A and a case study (M2 module) of group A. (a) A comprehensive view of survival analysis features of group A. The first column represents the predictive ability for the survival of genes in group A modules. The second column represents the predictive ability of the survival of ncRNA-regulated modules in group A. Two columns were the -log10(*P*) of survival analysis. The numbers were colored based on the adjacent color map. The details of these results for the M2 module are shown in (b) and (c). (b) Subnetwork of the predictive ability for the survival of genes of the M2 module. Nodes are colored as stage III COAD-DEGs, stage IV COAD-DEGs, or overlap genes of stage III COAD-DEGs and stage IV COAD-DEGs. Node size is shown according to its network degree. The differences in the survival times were analyzed using the log rank test. The *P* value for survival analysis was 0.01944. (c) Subnetwork and the predictive ability for survival of genes and ncRNAs of the M2 module. Nodes are colored as stage III COAD-DEGs, stage IV COAD-DEGs, overlap genes of stage III COAD-DEGs and stage IV COAD-DEGs, and pivot miRNA and pivot lncRNA of the M2 module. Node size is shown according to its network degree. The differences in the survival times were analyzed using the log rank test. The *P* value for survival analysis was 5.0175*E*-05.

In order to better understand the role of ncRNA posttranscriptional regulation in COAD progression, the genes of the 31 ncRNA-regulated modules mentioned above were extracted separately, and the predictive ability of COAD patient survival for each of these genes were evaluated. It was discovered that the predictive ability of 17 out of the 31 modules for COAD patient survival had improved compared to the genes of modules (Figure 3). For example, module 2 (M2) covers three lncRNAs, 17 miRNAs, and 60 mRNAs composed of both stage III and stage IV DEGs (Table 1). Notably, 19 out of 20 ncRNA regulators were supported by sufficient evidence (PubMed ID in Table 1) that they were involved in colorectal cancer (CRC) progression and metastasis (details in Supplementary [Supplementary-material supplementary-material-1]). Survival association was confirmed for the genes of M2 in three other independent cohorts of colorectal cancer patients (Table 1). The predictive ability for survival analysis was defined by -log10(*P*), where *P* was the statistical significance of survival analysis. The predictive ability of M2 increased from 1.70 without the ncRNAs to 4.30 with the ncRNAs, indicating that the ncRNAs are essential for M2 and contribute to a COAD patient's survival. In addition, we used three independent datasets of GSE29623, GSE39582, and GSE17536 to verify the contribution of 31 modules to COAD prognosis. The sample sizes were 65, 579, and 177, respectively. Almost all of the 31 modules were found to be significantly associated with the overall survival of COAD patients, except module 11 in GSE17536 (*P* = 0.42) and module 39 in GSE29623 (*P* = 0.08) (Supplementary [Supplementary-material supplementary-material-1]).

**Figure 4:**
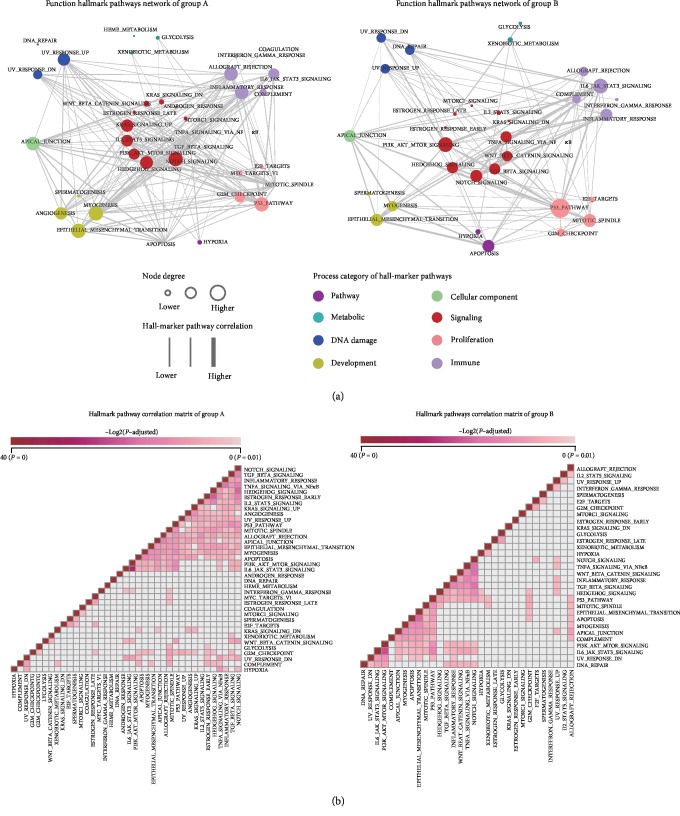
The function hallmark pathway network and hallmark pathway correlation matrix for group A and group B modules. (a) The function hallmark pathway network of group A and group B. Nodes in different colors stand for different categories of hallmark pathways. The node size indicates the degree of the nodes. The width of the edges indicates the correlation of hallmark pathways. (b) The hallmark pathway correlation matrixes of group A and group B. The correlation index matrix shows the similarity between each pair of hallmark pathways. The elements in the correlation matrix were the -log 2(*P*) of hallmark pathway correlation analysis.

Sequentially, the 31 COAD-related ncRNA regulatory modules were divided into two groups, in accordance to the result of the above survival analysis: group A (17 modules)—predictive ability for survival of ncRNA regulatory modules had improved compared to that of the genes and group B (14 modules)—the rest of the ncRNA-regulated modules. Then, the hallmark annotation enrichment analysis was applied to the modules of the two groups. If two hallmark pathways were significantly enriched by the same COAD-related ncRNA-regulated modules, then there may be links between the said two hallmark pathways during the process of COAD progression. Based on this assumption, a function hallmark pathway network was constructed for group A and group B modules, respectively (Figure 4). Finally, two hallmark pathway networks were obtained. The group A hallmark pathway network included 37 hallmark pathways and 207 edges (*P* < 0.01), and the group B hallmark pathway network included 31 hallmark pathways and 111 edges (*P* < 0.01).

Comparing the two hallmark pathway networks of group A and group B, it was found that the group A hallmark pathway network had closer interactions compared to that of group B (Figure 4). These close interactions in the group A hallmark pathway network were mainly concentrated in the pathways such as “APICAL_JUNCTION,” “EPITHELIAL_MESENCHYMAL_TRANSITION,” “MYOGENESIS,” and “P53_PATHWAY.” In the group A hallmark pathway network, the pathways which were significantly enriched by multiple COAD-related ncRNA regulatory modules and also had close interactions with other pathways may have an important role and are closely related to ncRNA posttranscriptional regulations during the process of COAD progression. These hallmark pathways were regarded as the core hallmark pathways (Supplementary [Supplementary-material supplementary-material-1]). Since core hallmark pathways closely interacted with each other, COAD-related ncRNA regulatory modules that were significantly enriched in the core hallmark pathways may need more regulatory elements to regulate the varied functions of these modules. Therefore, the integration of the ncRNA regulation information into these modules is necessary to elucidate ncRNA posttranscriptional regulation of COAD progression, and it could improve the sensitivity of the prognosis with these ncRNA regulatory modules.

The results indicated that the ncRNA-regulated modules which enriched the hallmark pathways had closer interactions with each other and were more concentrated in the core hallmark pathways. The integration of the ncRNA regulation information into these modules is very important. The above results suggest that ncRNAs can regulate the core hallmark pathways of COAD progression, and it is achieved through the regulation of these complex functions of COAD-related ncRNA regulatory modules.

## 4. Discussion

Given the high incidence of global COAD cases and increased mortality due to distant metastasis, it is of paramount importance to identify the novel regulatory pathways involved in COAD growth and metastasis. In recent years, a growing amount of research has shown that not only some important genes but also ncRNAs play key roles in the process of COAD progression [11, 12]. Presently, most of the researches only focus on certain gene families or miRNA families [11]. A comprehensive study of genes and essential ncRNA regulation in posttranscriptional level properties for COAD progression is still lacking. Therefore, a multidimensional integration strategy was used based on gene, miRNA, and lncRNA expression profiling, protein-protein interactions (PPIs), and posttranscriptional regulation data to identify biologically meaningful gene modules and their ncRNA regulators involved in the COAD progression.

Previous evidences indicate that ncRNAs could play a critical role in the regulation of cellular processes such as cell growth, apoptosis, and cancer progression and metastasis in colorectal cancer [11, 12]. The combination of Pivot and survival analysis detected the ncRNAs which regulated crosstalk modules across the stages of COAD. This resulted in 31 COAD-related modules regulated by ncRNAs. It was discovered that the predictive ability for COAD patient survival of 17 out of 31 COAD-related ncRNA regulatory modules had been improved compared to that of the same module genes without ncRNAs. In total, these 17 modules (said group A) contained 62 ncRNAs, which included 45 miRNAs and 17 lncRNAs.

Seven (XIST, AC003092.1, HCG18, CTD-2020K17.1, HOXA11-AS, RP11-452F19.3, and SBF2-AS1) of 17 lncRNAs belonged to two or a maximum of five modules of group A, of which XIST belonged to five modules. XIST significantly increases its expression in both CRC tissue sample and CRC cells and promotes CRC cell proliferation by the miR-132-3p/MAPK1 axis [35]. HCG18 promotes the growth and invasion of CRC cell via miR-1271/MTDH/Wnt/*β*-catenin signaling [36]. High expression of HOXA11-AS is a risk factor for distant metastasis and poor clinical outcomes in numerous tumors [37].

Also, 14 of 45 miRNAs belonged to two or a maximum of four modules of group A, of which five miRNAs (miR-30b-5p, miR-34c-3p, miR-103a-3p, miR-362-3p, and miR-590-3p) belonged to three modules and miR-130b-3p belonged to four modules. miR-130b-3p has been reported to play particularly significant roles in cancer progression [38]. A study discovered that miR-30b-5p is a tumor suppressor in CRC through the USP22/Wnt/*β*-catenin signaling axis [39]. miR-103a can inhibit the proliferation, migration, and tumor growth and metastasis in CRC cells [40], and miR-103a-3p overexpresses in CRC [41]. Overexpressed miR-362-3p influences cell cycle arrest and reduces cell viability and proliferation in CRC cells, which is associated with the recurrence of CRC [42]. miR-590-3p accelerates cellular proliferation and metastasis via targeting the Hippo pathway, and it can predict worse clinical outcomes of CRC patients [43].

There were common ncRNAs between group A and group B. Seven lncRNAs (RP11-452F19.3, AC005537.2, CDKN2B-AS1, SBF2-AS1, RP11-399K21.11, ENTPD1-AS1, and SNHG7) and 29 miRNAs including miR-362-3p and miR-590-3p mentioned above were unique in group A. SNHG7 can promote the proliferation, migration, and invasion and inhibit apoptosis in lung cancer, gastric cancer, or brain cancer [44–46].

Since they regulate the crosstalk modules across the stages of COAD, these 62 ncRNAs are likely to be significant biomarkers for diagnosis, recurrence, metastasis, and prognosis, as well as treatment response. It is reported that XIST expression is correlated with tumor size, N1, M1, and III+IV stages of CRC, and it can be an independent prognostic biomarker for CRC patients [47]. Studies have found that miR-103a-3p, miR-141, miR143, and miR-193-3p are associated with CRC diagnosis [11, 41]. It was also discovered that high expression levels of miR-141 and miR-181c, are major factors for prognosis, malignant potential, and CRC recurrence [48]. Low expression levels of miR-362-3p [42] and miR-130b [48] are prognostic in nature. Also, downregulated miR-106b-5p was negatively associated with lymph node metastasis [49]. Upregulation of let-7f-5p [50] and XIST [51] enhances chemotherapeutic resistance in CRC.

In addition, with the exception of ncRNA posttranscriptional level, further efforts should be committed to the study of the remaining regulatory elements, such as transcription factor, DNA methylation, and copy number variation, as they may also contribute to the discovery of more detailed molecular mechanisms and provide theoretical guidance for biological research in the future.

## 5. Conclusion

In summary, the study introduced a multidimensional integration strategy to identify gene modules that are biologically relevant, along with their ncRNA regulators involved in COAD progression and related to the overall survival of COAD patients. The ncRNAs which increase the predictive ability of overall survival are essential for the crosstalk modules across COAD stages. Both the modules and the ncRNAs have potential diagnostic and prognostic value.

## Figures and Tables

**Table 1:** Statistics about ncRNA-regulated module 2.

lncRNA (PMID)	miRNA (PMID)	Gene	*P* value^&^	*P* value^#^	*P* value^∗^ (dataset)
LINC00630 (28473661, 25908452), RP11-452F19.3, XIST (31452526, 28730777, 29504606)	Let-7f-5p (29805607), miR-7-5p (30867755), miR-18a-5p (30458288), miR-18b-5p (30458288), miR-26b-5p (28640257), miR-103a-3p (30458288), miR-106b-5p (30013364), miR-122-5p (28177881), miR-130a-p (28849155), miR-140-5p (31011255), miR-155-5p (2947100), miR-196a-5p (30621631), miR-301a-3p (30362160), miR-301b-3p (20132431), miR-362-3p (23280316), miR-590-3p (28938537), miR-590-5p (27735951)	A2M, ABL1, ACACB, ACVR2A, ACVR2B, ADCY2, ADSSL1, AGTR2, ATIC, BAMBI, BMP2, BMPR1A, BMPR1B, BMPR2, CACNA1C, CACNA1D, CACNA1F, CACNA1G, CACNA1H, CACNA1S, CACNA2D1, CACNB1, CACNB2, CACNB3, CACNB4, CDK5, CDT1, CHRD, CHRM5, CHUK, CLOCK, CRMP1, CRY1, CRY2, CSF2, CSNK1E, CXCR4, DPYSL3, DRD5, E2F1, EPHA3, EPHA5, EPHA6, EPHA7, F2RL3, FANCD2, FANCI, FOXO1, FOXO3, FST, FZD4, FZD5, FZD7, FZD8, GPER1, HGF, HPRT1, HRH4, HTR2B, IKBKB, INS-IGF2, IRS2, LPAR1, LPAR3, MCHR1, MED1, MED21, MED23, MED27, MED4, MET, MRE11A, MTNR1B, MYD88, NCOA1, NOG, NPAS2, P2RY13, PCNA, PDE4A, PDE4B, PDE4C, PDE4D, PER1, PER2, PIK3CB, PIK3R1, PLCG1, POMC, PPARGC1A, PRKAA1, PRKAA2, PRKAB1, PRKAB2, PRKACA, PRKACB, PRKAG1, PRKAG2, PRKAG3, PSMD11, PSMD6, PTGS2, QRFPR, RAC1, RAD17, RAD21, RAD50, RAD51C, RBBP8, S1PR1, S1PR2, S1PR3, SMAD2, SMAD5, SMAD9, SMC1B, SMC3, SMURF1, SMURF, SP1, SPO11, STAG2, STAT3, STK11, SYCE1, SYCE2, SYCP1, SYCP2, SYCP3, TEX12, TGFB3, TGFBR1, TGFBR2, TIMELESS, TOPBP1, UTS2R, WNT1, WNT2, WNT3, WNT3A, WNT5A, YES1	5.02*E*-05	0.0194	0.0015 (GSE29623), 2.20*E*-16 (GSE39582), 2.20*E*-16 (GSE17563)

*P* value is used to measure the overall survival of COAD patients by Kaplan-Meier analysis. & and # stand for the *P* value of M2 with or without ncRNA regulators from TCGA data and ∗ stands for the *P* value of M2 without ncRNA regulators in three other independent datasets from GEO. The numbers in the parentheses are PubMed IDs (PMID).

## Data Availability

The RNA-seq and clinical data of COAD in this study were obtained from TCGA (https://portal.gdc.cancer.gov/), and three validation datasets of GSE29623, GSE39582, and GSE17536 were from Gene Expression Omnibus (GEO) (https://www.ncbi.nlm.nih.gov/geo/).

## Supplementary Materials

Supplementary Materials Supplementary Table S1: statistic information about stage III-related functional modules. Supplementary Table S2: statistic information about stage IV-related functional modules. Supplementary Table S3: statistics about 31 ncRNA regulatory modules. Supplementary Table S4: function annotation of ncRNAs in module 2. Supplementary Table S5: statistics about core hall-marker pathways.
