# Supplementary material for: Contributions of Gene Modules Regulated by Essential Noncoding RNA in Colon Adenocarcinoma Progression
Source: Biomed Res Int. 2020 Mar 20;2020:8595473. doi: 10.1155/2020/8595473 (PMC7128050; doi:10.1155/2020/8595473)
Supplement: Supplementary Materials — Supplementary Table S1: statistic information about stage III-related functional modules. Supplementary Table S2: statistic information about stage IV-related functional modules. Supplementary Table S3: statistics about 31 ncRNA regulatory modules. Supplementary Table S4: function annotation of ncRNAs in module 2. Supplementary Table S5: statistics about core hall-marker pathways. [file 8595473.f1.zip › Supplementary Table S1 2 4 5.doc]

Supplementary TABLE S1: Statistic information about Stage III -related functional modules.
No. of Module	Score from MCODE	Count of Nodes	
1	33.88280375	44	
2	24.14873376	63	
3	25.7551594	82	
4	19.83592848	60	
5	24.50355999	32	
6	20	20	
7	16.04265174	24	
8	15.39881375	50	
9	13.18840602	143	
10	8.496386946	12	
11	10.62437565	73	
12	10	11	
13	10.92820609	9	
14	15.89516627	38	
15	5.875	8	
16	10.71277989	10	
17	6.753968254	9	
18	6	7	
19	13.26794998	8	
20	5	6	
21	8.227461133	103	
22	7.478787879	8	
23	6.382395662	130	
24	3.822222222	6	
25	9.090634144	57	
26	3.996825397	6	
27	4.630952381	5	
28	4	5	
29	8.139393939	6	
30	6.934148497	33	
31	3.657142857	5	
32	2.82	5	
33	4.460606061	6	
34	7.450532897	37	
35	2.893650794	7	
36	1.81462585	7	
37	5.561395588	20	
38	5.666666667	4	
39	5.785714286	4	
40	4.613095238	4	
41	3.107142857	4	
42	2.791117216	5	
43	3	4	
44	4.208936467	12	
45	11.07608746	7	
46	10.33643975	7	
47	6.060015742	11	
48	4.975968598	17	
49	4.205128205	4	
50	3	4	
51	2.85	4	
52	2.7	4	
53	3.673431514	14	
54	20.22392483	5	
55	20.59493635	3	
56	17.09851552	3	
57	9.581585082	3	
58	7.987123987	3	
59	6.685067155	5	
60	6.017253507	7	
61	5.666666667	3	
62	5.733333333	5	
63	5.666666667	3	
64	5.857142857	3	
65	5.121021957	19	
66	4.596560847	3	
67	4.398297491	3	
68	4.480886263	3	
69	3.857936508	3	
70	4	3	
71	3.311111111	3	
72	2.451851852	3	
73	3	3	
74	3	3	
75	2.8	3	
76	2.8	3	
77	3	3	
78	1.928571429	3	
79	2.133333333	3	
80	2	3	
81	2	3	
82	2	3	
83	10.65558126	4	

Supplementary TABLE S2: Statistic information about Stage IV -related functional modules.
No. of Module	Score from MCODE	Count of Nodes	
1	24.30977	36	
2	23.84288	68	
3	24.32788	63	
4	20.68829	34	
5	20.28812	67	
6	11.57292	16	
7	16.68001	40	
8	16.01813	68	
9	11.48526	23	
10	10.54012	28	
11	13.54001	86	
12	12.81442	48	
13	7.841975	9	
14	9.470597	65	
15	10.5253	18	
16	11.90211	22	
17	5.636364	11	
18	6.45772	15	
19	7	8	
20	8.05209	32	
21	11.10096	19	
22	6.875	8	
23	6	7	
24	4.95	9	
25	5	6	
26	12.15252	16	
27	3.822222	6	
28	7.232515	41	
29	6.233575	26	
30	8.225564	7	
31	8.525028	62	
32	3.996825	6	
33	4.761905	5	
34	6.425662	21	
35	3.813333	5	
36	2.82	5	
37	2.82	5	
38	4.6013	34	
39	6.041703	13	
40	5.666667	4	
41	4.648168	4	
42	4.613095	4	
43	3.647619	4	
44	3.402778	4	
45	3.133333	5	
46	3	4	
47	2.04	5	
48	5.727415	27	
49	7.126838	4	
50	4.880952	4	
51	3.036467	10	
52	5.093723	23	
53	19.77121	3	
54	18.16516	3	
55	12.33571	3	
56	7.502165	7	
57	6.870699	3	
58	6.685581	3	
59	5.808658	3	
60	5.547894	3	
61	5.2	5	
62	5.290452	3	
63	3.311111	3	
64	3.079365	3	
65	3	3	
66	3	3	
67	3	3	
68	2.8	3	
69	2.8	3	
70	3	3	
71	2.7	3	
72	2.046118	3	
73	2	3	
74	2	3	
75	2	3	
76	2	3	
77	2	3	
78	1.892857	3	
79	2	6	

TABLE S4: Function annotation of ncRNAs in Module 2.
Regulator 	PMID	Description	
LINC00630	25908452,
28473661	commonly dysregulated in colorectal cancer;
overexpression increased cell proliferation and metastasis in  NSCLCs.	
XIST	31452526,
28730777,
29504606	XIST Plays a Critical Role in Predicting Clinical Prognosis and Progression of Colorectal Cancer; 
Long non-coding RNA XIST functions as an oncogene in human colorectal cancer by targeting miR-132-3p; 
lncRNA XIST can be an independent risk factor for colorectal cancer prognosis.	
let7-f-5p	29805607	Upregulation of let-7f-5p promotes chemotherapeutic resistance in colorectal cancer by directly repressing several pro-apoptotic proteins.	
miR-7-5p	30867755	miR-7-5p inhibits CRC proliferation and migration by targeting KLF4, which suggests that miR-7-5p is a potential molecular target for the treatment of human CRC.	
miR-18a-5p	30458288	Seven miRNAs (miR-103a-3p, miR-127-3p, miR-151a-5p, miR-17-5p, miR-181a-5p, miR-18a-5p and miR-18b-5p) were significantly overexpressed in CRC
	
miR-18b-5p	30458288	Seven miRNAs (miR-103a-3p, miR-127-3p, miR-151a-5p, miR-17-5p, miR-181a-5p, miR-18a-5p and miR-18b-5p) were significantly overexpressed in CRC
	
miR-26b-5p	28640257	miR-26a and miR-26b might be implicated in cancer progression via their target gene FUT4, suggesting their potential usage in CRC treatment	
miR-103a-3p	30458288	Seven miRNAs (miR-103a-3p, miR-127-3p, miR-151a-5p, miR-17-5p, miR-181a-5p, miR-18a-5p and miR-18b-5p) were significantly overexpressed in CRC compared with NCs.	
miR-106b-5p	30013364	Our findings revealed that miR-106b-5p inhibits CRC metastasis by upregulating CTSA expression, which may lead to novel therapeutic strategies for CRC patients.	
miR-122-5p	28177881	miR-122-5p may be as potentially biomarker for discriminating colon cancer from rectal cancer.	
miR-130a-p	28849155	MicroRNA-130a is upregulated in colorectal cancer and promotes cell growth and motility by directly targeting forkhead box F2.	
miR-140-5p	31011255	overexpression of miR-140-5p reversed the effect of CASC19 on cell proliferation and tumor migration in CRC	
miR-155-5p	2947100	MiR-155-5p controls colon cancer cell migration via post-transcriptional regulation of Human Antigen R.	
miR-196a-5p 	30621631	miR-196a-5p promotes metastasis of colorectal cancer via targeting I¦ÊB¦Á.	
miR-301a-3p	30362160	Overexpression of miR-301a-3p promotes colorectal cancer cell proliferation and metastasis by targeting deleted in liver cancer-1 and runt-related transcription factor 3.	
miR-301b-3p	20132431	miR301b-3p were over-expressed in 3 matched cases of CRCs without metastases to lymph nodes.	
miR-362-3p	23280316	MiRNA-362-3p induces cell cycle arrest through targeting of E2F1, USF2 and PTPN1 and is associated with recurrence of colorectal cancer.	
miR-590-3p	28938537	MiR-590-3p promotes proliferation and metastasis of colorectal cancer via Hippo pathway.	
miR-590-5p	27735951	MiR-590-5p inhibits colorectal cancer angiogenesis and metastasis by regulating nuclear factor 90/vascular endothelial growth factor A axis.	

 

Supplementary Table S5. Statistics about core hall-marker pathways.
Hallmark name	Process Category	Description	Count of modules	
APICAL_JUNCTION	cellular component	apical junction complex consisting of adherens and tight junctions	9	
ANGIOGENESIS	development	blood vessel formation	5	
MYOGENESIS	development	muscle differentiation	8	
EPITHELIAL_MESENCHYMAL_TRANSITION	development	epithelial mesenchymal transition	9	
UV_RESPONSE_UP	DNA damage	UV response: upregulated genes	4	
ALLOGRAFT_REJECTION	immune	allograft rejection	7	
IL6_JAK_STAT3_SIGNALING	immune	IL6 STAT3 signaling during acute phase response	10	
INFLAMMATORY_RESPONSE	immune	inflammation	8	
APOPTOSIS	pathway	programmed cell death; caspase pathway	8	
MITOTIC_SPINDLE	proliferation	cell cycle progression: mitotic spindle assembly	16	
P53_PATHWAY	proliferation	p53 pathway	12	
ESTROGEN_RESPONSE_EARLY	signaling	early estrogen response	8	
HEDGEHOG_SIGNALING	signaling	Hedgehog signaling	9	
KRAS_SIGNALING_UP	signaling	KRAS signaling, upregulated genes	6	
NOTCH_SIGNALING	signaling	Notch signaling	11	
PI3K_AKT_MTOR_SIGNALING	signaling	PI3K signaling via AKT to Mtorc1	10	
TGF_BETA_SIGNALING	signaling	TGF beta signaling	8	
TNFA_SIGNALING_VIA_NFKB	signaling	TNFA signaling via NFkB	10	
IL2_STAT5_SIGNALING	signaling	IL2 STAT5 signaling	7	
